# Skin Tissue Engineering: Application of Adipose-Derived Stem Cells

**DOI:** 10.1155/2017/9747010

**Published:** 2017-02-27

**Authors:** Agnes S. Klar, Jakub Zimoch, Thomas Biedermann

**Affiliations:** ^1^University Children's Hospital Zurich, Tissue Biology Research Unit, Zurich, Switzerland; ^2^Children's Research Center, University Children's Hospital Zurich, Zurich, Switzerland

## Abstract

Perception of the adipose tissue has changed dramatically over the last few decades. Identification of adipose-derived stem cells (ASCs) ultimately transformed paradigm of this tissue from a passive energy depot into a promising stem cell source with properties of self-renewal and multipotential differentiation. As compared to bone marrow-derived stem cells (BMSCs), ASCs are more easily accessible and their isolation yields higher amount of stem cells. Therefore, the ASCs are of high interest for stem cell-based therapies and skin tissue engineering. Currently, freshly isolated stromal vascular fraction (SVF), which may be used directly without any expansion, was also assessed to be highly effective in treating skin radiation injuries, burns, or nonhealing wounds such as diabetic ulcers. In this paper, we review the characteristics of SVF and ASCs and the efficacy of their treatment for skin injuries and disorders.

## 1. Introduction

Although a tremendous progress has been made, large full-thickness skin defects are still associated with mortality due to a low availability of donor skin areas.

Autologous cultured epidermal autografts (CEA) were first to be used as an epidermal substitute; however, their functional and esthetic results were unsatisfying due to graft contracture, scars, and infections [1–3]. The addition of a cellular dermal component to those skin substitutes resulted in an improved function and esthetic appearance [4–6]. These improvements are due to the presence of fibroblasts, which produce extracellular matrix (ECM) proteins such as collagen, elastin, laminin, and fibronectin that provide a mechanical stability to the dermis and regulate the function of cells in the epidermis, keratinocytes and melanocytes. Recently, dermo-epidermal tissue-engineered skin substitutes (DESS) emerged as an alternative in the treatment of deep burns and various skin-related disorders mimicking a near-natural skin appearance with regard to the skin texture [7], color [8, 9], and mechanical properties [6].

Due to the shortage of skin donor sites following large full-thickness skin injuries, new cell sources have been exploited for skin tissue regeneration. Adipose tissue represents an abundant and easily accessible source of adult stem cells for translational clinical approaches including skin tissue engineering [10, 11]. The stromal vascular fraction (SVF) is a freshly isolated heterogeneous cell population, which is derived from excised adipose tissue or liposuctions. The SVF may be further used for selection and expansion of an adherent population, so-called adipose-derived stem cells (ASCs). The ASCs are characterized by the expression of specific markers and their ability to differentiate into cells from meso-, ecto-, and endodermal lineages. However, recently introduced new nomenclature, isolation protocols, culture techniques, and differentiation methods lack standardization and may lead to misunderstandings.

In this paper, we review the characteristics of SVF and ASCs and summarize current developments regarding the in vitro skin models and approaches in direction of a complete full-thickness skin replacement. Clinical applications of SVF and ASCs in reconstructive surgery are also discussed to reveal their potential in regenerative medicine.

## 2. Characterization of the Human Stromal Vascular Fraction (SVF)

Despite different types of adipose tissue, its cell composition is rather constant. Mature adipocytes made up most of the population of the human body fat but they are also accompanied by preadipocytes, fibroblasts, stem cells, various progenitors, endothelial cells, and immune cells.

The SVF is a highly heterogeneous population with subpopulations' percentages depending on the adipose tissue depot site and the isolation procedure. It has been reported that stem and progenitor cells in the SVF usually amount up to 3% of the entire cell population, 2,500-fold more than the stem cell frequency in bone marrow [12]. A single subcutaneous liposuction yields approximately 0.5–2.0 × 10^6^ [13] nucleated SVF cells per gram of adipose tissue containing stem cells in the range of 1 to 10% [14]. In contrast, a bone marrow transplant delivers only approximately 6 × 10^6^ nucleated cells [13], 0.001–0.01% of which are stem cells [15].

Comparing to the bone marrow, the mononucleated fraction of the SVF is also richer in stromal cells (15–30% of all cells). Other lineages present in the SVF are endothelial cells (10–20%), granulocytes (10–15%), monocytes (5–15%), lymphocytes (10–15%), pericytes (3–5%), hematopoietic stem, and progenitor cells (<0.1%) [16–18]. Up to date, no unique antigen but only a combination of different markers has been used to identify distinct SVF subpopulations.

The golden standard to distinguish the stromal component of the SVF from the cells of hematopoietic origin is CD45 (Leukocyte Common Antigen) antigen [19–21]. CD31 (PECAM-1) can be used for endothelial cells and endothelial progenitors. Moreover, the well-known CD34 marker for primitive blood- and bone marrow-derived progenitor cells has been successfully used for the SVF subpopulations [22]. Abundance of CD34^+^ cells in the SVF fraction depends again on the adipose tissue location and method of isolation but it has been shown that this subpopulation represents at least 20% of the freshly harvested SVF [22, 23]. Other popular markers used in combination to characterize the SVF are CD13, CD90, CD105, CD73, CD10, and CD29 [19, 24]. Nevertheless, the published data on the surface marker expression of SVF and ASCs is inconsistent. Additionally, investigators use varying nomenclature for isolated cell populations, generating further confusion in the literature.

## 3. Adipose-Derived Stem Cells (ASCs)

The BMSCs became a golden standard in regenerative medicine. While they still remain at the prime position for treatment of several diseases, they carry disadvantages such as painful isolation procedure, need for general anesthesia, and low cell yields. In comparison, the ASCs can be obtained in large quantities during a single liposuction and without general anesthesia. Both stem cell populations, BMSCs and ASCs, are of mesenchymal origin and were shown to possess similar properties of self-renewal and abilities of multipotential differentiation [25–28].

The ASCs subset can be refined from the SVF after seeding and culturing on tissue culture plastic. Combination of washing steps, in vitro expansion, and serial passaging enables purification of this subset by elimination of nonadherent erythrocytes and hematopoietic cells. The obtained subset of adherent, spindle shaped cells is called adipose-derived stem cells (ASCs) and cultured under conditions similar to the ones used for BMSCs. Studies of Zuk and coworkers in 2001 and 2002 were first to characterize the multipotent character of the ASCs [29, 30].

Initially, ASCs cultures represent a heterogeneous population including “stromal” cells at various differentiation stages [29, 30]. Moreover, cultured ASCs dramatically change their phenotype and antigen expression during in vitro passaging. In general, ASCs can be characterized by antibodies recognizing the presence of the following antigens: CD73, CD90, CD105, and CD36. The CD36 surface antigen allows the distinction between ASCs and BMSCs [19, 31].

Manifesting aforementioned features and other beneficial characteristics, the ASCs are intensively investigated for use in translational and regenerative medicine. However, it is crucial to recognize the SVF as a primary source of ASCs and to distinguish between these two populations. However, a diverse nomenclature has been used in the literature leading to misunderstandings. Following terms have been used for cells selected from the SVF by plastic adherence: “adipose-derived adult stem cells,” “adipose-derived stromal cells,” “adipose stromal cells,” “adipose mesenchymal stem cells,” “processed lipoaspirate cells,” or “adipose-derived stromal/stem cells.” To avoid the misunderstandings by using these different terms, the International Federation for Adipose Therapeutics and Science (IFATS) recommends using the term “adipose-derived stem cells” to identify only the plastic-adherent, cultured, and in vitro expanded cell population and to distinguish it from the freshly harvested and noncultured SVF cells [24, 32, 33]. Therefore, in the present review we acknowledge these guidelines and follow their terminology accordingly.

## 4. Secretome of ASCs

Recently, the secretome of fat-derived ASCs drew much attention as a possible mechanism of their regenerative potential. Recent studies have demonstrated a low antigenicity and potent immunomodulatory effects of ASCs [34–37]. These observations make ASCs plausible candidates for allogenic cell therapies and eventually prompted their usage in solid organ transplants. Based on numerous studies, the ASCs were shown to secrete following factors into culture media: transforming growth factor *β* 1 (TGF-*β*1), tumor necrosis factor *α* (TNF-*α*), prostaglandin E2 (PGE2), granulocyte macrophage colony-stimulating factor (GM-CSF), and interleukins: 6, 7, 8, and 11 [38–40].

Several research groups have confirmed that administration of the ASCs is beneficial for angiogenesis [22, 41–43]. Further studies investigating the molecular mechanisms on this phenomenon have shown that factors secreted by ASCs such as vascular endothelial growth factor (VEGF), hepatocyte growth factor (HGF), basic fibroblast growth factor (bFGF), or angiopoietin-1 and -2 (Ang-1 and -2) play an important role in this process [42–44]. These findings are closely related to the studies performed on cardiac regeneration showing that VEGF and insulin growth factor 1 (IGF-1) secreted by ASCs contributed to increased tissue regeneration after myocardial infarction [45–47]. Similarly, the ASCs were demonstrated to promote skin tissue regeneration following injuries [48, 49].

Furthermore, the effects of ASCs on the central nervous system have been evaluated. Recent in vivo data demonstrated an important role of brain-derived neurotrophic factor (BDNF), nerve growth factor (NGF), and glial cell-derived neurotrophic factor (GDNF) in differentiation, protection, and survival of neurons [43, 68, 69].

## 5. Towards Clinical Application: Use of Human Adipose-Derived Stem Cells

### 5.1. Isolation of the SVF for Intraoperative Approaches

Once the material from liposuction is obtained, the SVF can be isolated manually or by automated devices. Manual isolation procedures became a standard procedure employed by many groups worldwide [70]. The adipose tissue is digested by collagenase, dispase, or trypsin. Dedicated mixtures of these and other enzymes are also commercially available. Steps that follow enzymatic digestion include subsequent washing, centrifugation, and filtration of obtained material. However, although useful in laboratory set-up, the manual procedure is far from ideal for many clinical applications. Despite the fact that some GMP-grade enzymes to isolate SVF for clinical trials are available on the market, there are other critical factors such as inconsistency in enzyme activity, endotoxin residues, other protease activities, and cell surface marker cleavage that significantly hamper their use. To address these issues, numerous automated devices have been developed [71–73]. The main goal in the development of those automated systems was to increase the concentration of isolated cells by procedure standardization. Most of the devices for the SVF isolation are based on a closed system of sterile containers where lipoaspirate is processed, washed, and concentrated. These devices not only obtain similar yields of cells as manual procedures but additionally the entire isolation can be completed in between 60 and 150 minutes [74, 75]. The major advantage of these medical devices is the possibility of an automated lipoaspirate processing at the patient's bedside for an intraoperative generation of autologous cells for a one-step surgical procedure. This fact may play an important role in popularizing adipose tissue cell therapies in clinics. The most important question, however, is whether automated isolation procedures can compete with the standard operator-based manual cell isolation. Studies comparing these two approaches showed superiority of the former in terms of cell yield, clonogenicity, phenotype, and differentiation potential of the isolated cells [76].

### 5.2. Differences of Adipose Tissue at Different Sites of the Body

In mammals, adipose tissue exists in two main types: white adipose tissue (WAT) and brown adipose tissue (BAT). WAT stores energy mainly in the form of triacylglycerols and BAT generates body heat. Functional BAT has been located in humans in the neck, mediastinal, supraclavicular, and interscapular body regions. WAT is found throughout the body as subcutaneous and visceral adipose tissues. Recently, Baglioni et al. observed dramatic differences in proliferation and adipogenic potential between the two ASCs populations isolated from abdominal subcutaneous and visceral omental adipose tissue with the first having a significantly higher growth rate and adipogenic potential than the latter [77].

Beside the harvest location, other factors such as age, body mass index, and gender influence the material collected by liposuction [78]. Aust et al. examined female patients and described a negative correlation between stem cell concentrations obtained in liposuction with body mass index but found no relation with age [79].

Jurgens and coworkers demonstrated that also the fat harvest site affects the yield of ASCs [80]. The group reported much higher yield of ASCs from the SVF in abdominal subcutaneous fat than in hip/thigh subcutaneous fat. However, the total amount of nucleated cells per volume or the ASCs proliferation and their differentiation capacities were not dependent on the tissue-harvesting site. The authors concluded that the abdomen seems to be preferable to the hip/thigh area for harvesting subcutaneous fat, in particular when SVF cells should be used intraoperatively in one-step surgical procedures.

The same group compared the effects of the surgical harvesting procedures such as resection, tumescent liposuction, and ultrasound-assisted liposuction in relation to the yield of SVF cells [14]. They reported that yield and growth characteristics of ASCs were affected by the type of surgical procedure used for harvesting, with ultrasound-assisted liposuction yielding the lowest number of proliferative cells. In summary, the published data show that more viable adipose-derived stem cells can be obtained from abdominal fat than from other body parts.

## 6. Skin Tissue Engineering

Large full-thickness skin defects resulting from burns, soft tissue trauma, congenital giant nevi, disfiguring scars, or tumor resection remain major clinical problems to patients and physicians [81, 82]. Skin autografts can be used to cover skin injuries of less than 30% of the total body surface area (TBSA) during one operation. However, if full-thickness skin defects affect more than 30% TBSA, the donor site available for an autograft becomes limited, so that alternative skin coverage is required [83].

To overcome this limitation, cultured epidermal autografts (CEA) consisting of keratinocytes were developed to provide enough autologous skin for the patient [1]. However, the routine use of CEA was hampered by its high risk of recurrent open wounds, long-term fragility, and increased rates of scar contractures.

**Figure 1:**
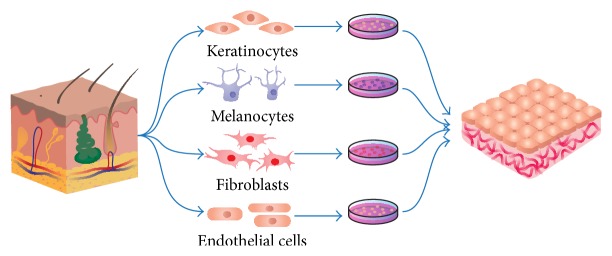
Development of a three-dimensional prevascularized dermo-epidermal skin substitute. Primary cells including epidermal keratinocytes, melanocytes, dermal fibroblasts, and endothelial cells can be isolated from a single skin biopsy. Dermal fibroblasts and endothelial cells are embedded into a collagen type 1 hydrogel to create a prevascularized dermal compartment. After they remodeled the collagen matrix, keratinocytes and melanocytes are then seeded onto it to create a pigmented epidermal layer.

Tissue-engineered dermo-epidermal skin substitutes (DESS) containing both epidermal and dermal layers have been developed by our group with the aim to produce large, near-natural skin substitute for the clinical use [7, 8, 10, 11, 83–87] (Figure 1). They demonstrate a strong resemblance to the natural skin with regard to its function and structure. Clearly, these are the most advanced and sophisticated skin products presently available for skin replacement.

Recently, we reliably reproduced the patient's own constitutive skin color by adding melanocytes to obtain pigmented DESS (pigmentDESS) [7, 8]. Hence, these substitutes do not only show an improved long-term aesthetic outcome but are also effectively protected against UV radiation.

### 6.1. Biomaterials in Use

Stem cells isolated from adipose tissue represent an attractive and valuable tool for regenerative skin engineering. Numerous in vitro and in vivo studies demonstrated their ability to differentiate into various skin cell lineages. Moreover, ASCs are recognized as a powerful source for skin regeneration because of their capability to secrete paracrine factors initiating tissue repair and to accelerate wound closure and promote skin regeneration instead of scar formation.

In this respect, a selection of the right biomaterial for the culture and differentiation of ASCs is of crucial importance [88]. It has to be compatible with the human body, support growth and expansion of skin cells, and finally resemble natural properties of the skin. Therefore, various scaffolds and biomaterials have been tested for the creation of artificial skin substitutes. Not surprisingly, many of them were also successfully applied in combination with ASCs or SVF. In the following, we describe a few of the most popular and commonly used biomaterials for skin replacement products.

Trottier et al. presented an interesting and, more importantly, successful concept of creation of skin substitutes without any use of external matrix or scaffold [89]. Their method is based on the endogenous production of the ECM components by different skin cells under specific culture conditions. After stimulation with ascorbic acid, stromal cells such as dermal fibroblasts or ASCs start an extensive production of ECM proteins [89, 90]. This leads to the formation of a cell sheet, which is strong enough to be manipulated and folded in order to additionally increase the thickness of a graft. The ASCs applied with this methodology have proven to be superior to previous efforts using dermal fibroblasts. The authors obtained satisfactory epidermal thickness and stratification. Further exploits led to the development of a three-layered skin substitute that also included a hypodermis.

Collagen type I of animal origin is one of the most commonly used polymers for the production of skin substitutes [91]. Its low immunogenicity and the excellent mechanical properties support the cell growth and make it a perfect scaffold material for skin tissue engineering. We have successfully utilized bovine type I collagen using the whole human SVF to develop a vascularized dermal component of the skin [10]. Prepared constructs were cultured under conditions promoting both the growth of stromal/dermal cells and capillary network formation by endothelial cells. This approach proved the possibility of prevascularization of collagen type 1 hydrogel-based skin substitutes in vitro. When these grafts were transplanted onto the back of immunocompromised rats, the preformed human capillaries present in the graft rapidly anastomosed (connected) to the rat vascular system allowing fast blood flow throughout the graft.

Similar approach was employed by Chan et al. in order to develop vascularized skin substitutes [92]. The authors, however, used various biomaterials within one skin substitute to drive the fate of the ASCs in different lineages. The cells seeded in a collagen type 1-based matrix turned into fibroblast-like dermal cells, whereas the same cells submerged into a PEGylated-fibrin-based layer developed into a blood capillary network. Additionally, the ASCs were differentiated into adipocytes in a third, collagen type 1-based layer of construct, forming the hypodermis.

Whereas Chan et al. used modified fibrin hydrogels only for the part of their substitutes, Monfort et al. based their skin substitutes solely on fibrin [93]. In their study the authors focused on the development of three-layered skin substitutes that included the epidermis, the dermis, and the hypodermis. The investigators used the ASCs in the hypodermal part, where they successfully differentiated them into adipocytes. The bioengineered hypodermis interacted with the upper layer of the substitute beneficially influencing the behavior of the epidermis in vitro.

The group of Kellar et al. developed a novel tropoelastin-based scaffold for skin substitutes [94]. In this study, tropoelastin, which is the precursor to elastin found in skin, was expressed in* E. coli* to produce large quantities of this protein following a skin scaffold development using electrospinning procedures. Subsequently, this biomimetic network was seeded with ASCs, cultured in vitro, and transplanted onto the SCID mice. The study demonstrated that the ASCs not only successfully colonized the scaffold, but also continued to grow. In vivo experiments showed a rapid wound closure and increased thickness of the epidermis as compared to the controls.

Some investigators went even further and examined the feasibility of a sodium carboxymethylcellulose scaffold for skin repair using ASCs [95]. Based on previous experiments confirming its compliance with requirements for a suitable scaffold, the authors evaluated its capability to support the cell growth. The group showed that only at certain concentration the new material is feasible for transplantation altering the wound healing. Although no acceleration of wound closure was observed, the ASCs promoted in vivo proliferation of the granulation tissue and epidermal stratification as compared to the control group.

This brief summary of various biomaterials that can be combined with ASCs or SVF showed that these cells are not different from other cells used for the development of skin substitutes. Nevertheless, the matrices derived from natural sources, like collagen or fibrin, have an advantage over synthetic scaffolds requiring more sophisticated production methods.

## 7. Clinical Use of Fat Autografts and Cell-Assisted Lipofilling for Plastic Skin Reconstruction

**Figure 2:**
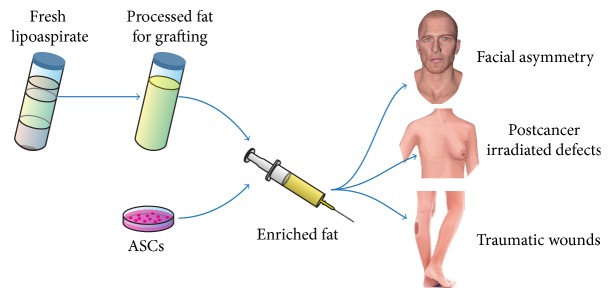
Examples of clinical application of autologous fat and adipose-derived stem cells (ASCs). Freshly isolated lipoaspirate is processed to obtain a fat graft. This can be applied to patients suffering, for example, from facial asymmetry, radiated defects, or traumatic wounds. The fat graft can be further enriched by adding freshly isolated SVF or cultured adipose-derived stem cells (ASCs).

The autologous fat grafting has been increasingly used in reconstructive and esthetic medicine [96] (Figure 2). For this purpose adipose tissue is aspirated from the patient's subcutaneous fat depots, purified, and then transplanted to the desired site of the body. Total fat grafting obtained by liposuction has been successfully applied as a soft tissue enhancement method for different defects due to disease, trauma, congenital defects, or the natural process of aging [96–99]. In general, fat grafting results in good to excellent short-term outcome, especially as it requires only minimally invasive (liposuction) harvesting procedure with low associated donor site morbidity. However, a single fat grafting is usually not sufficient and repeated fat transfer might be required. It is explained by the fact that fat grafting injections fill defects mostly with adipocytes, which eventually undergo apoptosis. This leads to scaring when fat grafts are incorporated more than 2 mm away from blood supply at the recipient site [100]. This results often in massive tissue resorption caused by poor neovascularization and leads to poor long-term outcome with 20–90% volume loss of applied fat [101–103]. Therefore, it would be ideal to support a fast vascularization of applied fat tissue to support its survival and take, eventually resulting in a better long-term outcome.

In 2006, Matsumoto et al. introduced the concept of a cell-assisted lipofilling (CAL) method in mice [104]. The authors used freshly isolated autologous SVF combined with the fat graft, which worked as a scaffold. A supplementation with SVF resulted in increased graft retention and enhanced microvasculature, confirming its clinical potential. Therefore, cell-assisted lipotransfer is considered to be superior to conventional autologous lipotransfer. Since then many investigators have applied CAL using the whole SVF or isolated ASCs in human trials [105].

In general, adipose stem cells are applied to enhance fat graft take, to heal difficult wounds with only poor blood supply, and to treat soft tissue damage (Table 1) [40, 106–109]. The positive effects of ASCs are attributed to the accelerated angiogenesis and lymphangiogenesis, reduced fibrogenesis, and inflammatory responses [38, 110–114]. Table 1 gives an overview of recent clinical applications of autologous fat and/or ASCs supplemented therapy. Unfortunately, there has been confusion in nomenclature concerning clinical therapies applying ASCs or SVF, as some studies used these two different terms interchangeably.

## 8. Conclusions

Adipose-derived stem cell therapy has gained tremendous interest in the area of skin repair and regeneration as a new paradigm for skin function restoration after injury. Compared to BMSCs, sufficient number of ASCs can be obtained from a relative small amount of adipose tissue during a minimally invasive harvesting procedure. Therefore, ASCs are recognized as an attractive substitute for BMSC for therapeutic applications.

However, apart from the numerous in vitro and in vivo studies demonstrating the high therapeutic potential of ASCs, the lack of standardized cell isolation methods as well as culture and differentiation protocols need to be overcome to advance the clinical utility of these cells. Moreover, additional safety studies and quality controls tests are necessary to translate scientific findings from basic science into the standard skin wound care plan in the clinic.

## Figures and Tables

**Table 1:** Clinical applications of autologous fat and SVF/ASCs.

	Medical condition	Study	Application	Total number of patients and sex if mentioned	Outcome
Soft tissue					

*Breast augmentation/reconstruction/ soft tissue defect*					

	Cosmetic breast augmentation	Yoshimura et al. 2008 [50]	Cell assisted lipotransfer (CAL) of SVF/ASCs and lipoinjection	40 (female)	Preliminary results suggest efficacy and safety

	Breast augmentation after breast implant removal	Yoshimura et al. 2010 [51]	Cell assisted lipotransfer of SVF/ASCs and lipoinjection	15 (female)	Very satisfactory outcome 12 months after application

	Breast augmentation	Kamakura and Ito [52]	Cell assisted lipotransfer of SVF and lipoinjection	20 (female)	Patient satisfaction was 75% and physician satisfaction 69%

	Breast augmentation	Wang et al. 2012 [40]	Cell assisted lipotransfer of ASCs/SVF and lipoinjection	18 (10 patients completed, 6 months' follow-up)	6-month postoperative, the breast volume is significantly increased and the breasts' contour is improved

	Breast reconstruction	Gentile et al. 2012 [53]	Cell assisted lipotransfer of SVF and lipoinjection	10 (out of total 23)	1 year postoperative, 63% maintenance of the contour restoring and of three-dimensional volume compared with the control patients treated with fat graft only

	Breast augmentation	Peltoniemi et al. 2013 [54]	Water assisted lipotransfer (WAL) enriched with SVF	10 (out of total 18 patients, females)	No advantage in SVF stem cell enrichment in cosmetic fat transplantation observed: breast augmentation by WAL alone was faster, cheaper, with lower risk of contamination, offered at least an equal take rate

	Healthy participants	Kølle et al. 2013 [55]	Fat grafting after liposuction enriched with ASCs	10 (females)	ASCs enriched fat grafts had significantly higher residual volumes; no serious adverse events were noted; procedure of ASCs-enriched fat grafting had excellent feasibility and safety

	Secondary breast reconstruction	Tissiani and Alonso 2016 [56]	Fat grafts enriched with SVF	11 (out of total 19, females)	SVF enriched fat grafts have proven to be safe in a 3-year follow-up

	Various including breast reconstruction, scarring, Parry-Romberg disease, gluteal soft tissue defect, pectus excavatum, polio infection sequel, and dermatofibromatosis	Tiryaki et al. 2011 [57]	Fat grafts enriched with SVF	29	Preliminary results suggest SVF enriched fat grafting was safe and may provide superior results compared to traditional fat grafting

	Burns sequelae and posttraumatic scars	Gentile et al. 2014 [58]	Fat grafts enriched with SVF	10 (out of total 30)	No complications in any patient; the results were lasting in all cases; all patients were satisfied with the resulting texture, softness, contour; MRI confirmed absence of cyst formation and microcalcification

	Systemic sclerosis	Granel et al. 2015 [59]	Autologous SVF injection in the finger of systemic sclerosis patients	12 (females)	6 month after procedure no severe adverse events occurred; four minor adverse events were reported and resolved spontaneously; significant improvement in hand disability and pain, Raynaud's phenomenon, finger oedema, and quality of life was observed

	Systemic sclerosis	Guillaume-Jugnot et al. 2016 [60]	Autologous SVF injection in the finger of systemic sclerosis patients	12 (female)	12 months after procedure a significant improvement of finger oedema, skin sclerosis, motion, strength of the hands, and of vascular suppression score was noted

Facial lipoatrophy/facial defects					

	Congenital or acquired facial tissue defects (Barraquer-Simons syndrome; Parry Romberg syndrome; traumatic; facial atrophy; lupus erythematosus)	Sterodimas et al. 2011 [61]	Lipografts enriched with SVF	10 (out of total 20)	Analysis of patient satisfaction in the first six months clearly demonstrated better results using SVF; by the 18-month evaluation, no statistical difference between the lipograft only or lipograft/SVF treatment in terms of patient satisfaction noted

	Progressive hemifacial atrophy (Parry-Romberg disease)	Castro- Govea et al. 2012 [62]	Cell assisted lipotransfer of SVF and lipoinjection	1 (male)	1 and 12 months postoperative evolution of patient was satisfactory; reduction of severe depression of the frontotemporal region, better volume, and symmetry provided

	Progressive hemifacial atrophy (Parry-Romberg disease)	Koh et al. 2012 [63]	Microfat grafting enriched with ASCs	5 (3 females, 2 males) (out of total 10, 5 females, 5 males)	Successful outcomes were evident in all 5 patients receiving microfat grafts and ASCs; survival of grafted fat was better than in patients receiving microfat grafts alone

	Progressive hemifacial atrophy (Parry-Romberg disease)	Chang et al. 2013 [64]	Fat grafts enriched with SVF	10 (out of total 20)	After 6 months fat survival and clinical improvement were greater with SVF-supplemented grafting than fat grafting alone

Wound healing					

*Radiation atrophy*					

	Therapy for side effects of radiation treatment with severe symptoms or irreversible function damage	Rigotti et al. 2007 [65]	Repeated lipoaspirate (SVF) injection	22 (females)	Clinical outcomes led to a systematic improvement or remission of symptoms in all evaluated patients, including otherwise untreatable patients exhibiting initial irreversible functional damage

*Ischaemic wounds*					

	Critical limb ischemia (CLI) patients with ischemic resting pain in 1 limb with/without nonhealing ulcers and necrotic foot	Lee et al. 2012 [66]	Intramuscularly injection of ASCs	15 (male)	6 months after application: significant improvement was noted on pain rating scales and in claudication walking distance; digital subtraction angiography showed formation of numerous vascular collateral networks across affected arteries

	Chronic ulcers of the lower limbs	Marino et al. 2013 [67]	Injection of SVF to the edges of ulcers	10 (3 females, 7 males) (out of total 20, 14 males, 6 females)	Reduction in diameter and depth of the ulcer, decrease in pain associated with the ulcer process; in six of 10 cases there was complete healing of the ulcer
